# An investigation of the frequency of bacteraemia following dental extraction, tooth brushing and chewing

**DOI:** 10.5830/CVJA-2012-016

**Published:** 2012-07

**Authors:** Breminand Maharaj, Yacoob Coovadia, Ahmed C Vayej

**Affiliations:** Department of Therapeutics and Medicines Management, University of KwaZulu-Natal, Durban, South Africa; Department of Medical Microbiology, University of KwaZulu-Natal, Durban, South Africa; Programme: Oral Health, Department of Health, KwaZulu-Natal, Durban, South Africa

**Keywords:** bacteraemia following dental procedures

## Abstract

**Abstract:**

We conducted a study to determine the frequency of bacteraemias following dental extraction and common oral procedures, namely tooth brushing and chewing, and the relationship between bacteraemia and oral health in black patients. Positive blood cultures were detected in 29.6% of patients after dental extraction, in 10.8% of patients after tooth brushing and in no patients after chewing. No relationship between the state of oral health, which was assessed using the plaque and gingival indices, and the incidence of bacteraemia was found. The duration of bacteraemia was less than 15 minutes. One patient had a positive blood culture prior to dental extraction; his oral health status was poor. Our study confirmed that bacteraemia occurs after tooth brushing.

Dental treatment has been regarded as a major cause of infective endocarditis, mainly because of the high frequency of bacteraemia after various oral procedures and the high recovery rate of viridans streptococci from the blood of patients with infective endocarditis.^1^-^3^ Awareness of the relationship between infective endocarditis and dental extraction dates back to 1909, when Horder noted the association between *Streptococcus viridans* in the oral cavity and infective endocarditis in patients with heart disease.^4^

Bacteria may invade the bloodstream after a wide variety of clinical procedures.^5^ Lewis and Grant postulated that healthy persons frequently have innocuous, transient bacteraemia and that the defective heart valve may trap and retain these organisms, resulting in infective endocarditis.^6^ Okell and Elliot noted streptococcal bacteraemia following dental extraction in 61% of their patients.^7^

Many investigators have assessed the incidence of transient bacteraemia following various oral procedures. The frequency of positive blood cultures has ranged from zero to 85% (mean: 40%) for dental extraction, from eight to 79% (mean: 35%) for dental scaling, from 36 to 88% (mean: 58%) for periodontal surgery, from seven to 50% (mean: 25%) for tooth brushing or irrigation, and from zero to 51% (mean: 38%) for chewing.^5^,^8^ Bacteraemia has been detected following flossing,^9^ procedures used for conservative dentistry,^2^ intra-oral suture removal,^10^ and endodontic treatment.^11^

Although viridans streptococci are the micro-organisms most frequently isolated in these studies, considerable differences in frequency, type and magnitude (colony counts per millimetre of blood) of post-procedure bacteraemia have been reported. This is mainly the result of diversities in the type of surgical procedures (e.g. single vs multiple dental extraction), time of blood sampling, volume of blood cultured, and the methods used to isolate and identify the micro-organisms, which hinder the interpretation and comparison of results. The reports published before the 1960s may also have underestimated the incidence of transient bacteraemia, since no refined anaerobic culture techniques were available.^12^

Because some of the earlier investigations on antibiotic prophylaxis had failed to show eradication of bacteria, and the state of oral health had not been controlled in these studies,^13^,^14^ we decided that it would be important to rule out the possible influence of oral health on post-extraction bacteraemia. Also, the frequency of bacteraemia following other common oral procedures, which have been recorded to produce bacteraemia, had not been evaluated in black patients. This study was designed to determine:

• the relative frequency of bacteraemia following tooth extraction, tooth brushing and chewing in black patients
• whether the state of oral health influenced the occurrence of bacteraemia after these procedures
• the duration of bacteraemia after these procedures.

## Methods

Adult black patients attending the Dassenhoek Dental Clinic in Marianhill near Durban were included in the study, after informed consent had been obtained. They were healthy, had no history of cardiovascular disease and had not received antibiotics in the previous two weeks.

Any patient found to have a dental abscess was excluded. In addition, in the extraction part of the study, any patient who needed more than one tooth extracted or required general anaesthesia was excluded.

The age and gender of each patient was recorded. The oral health status was evaluated by clinical examination and calculation of the plaque and gingival index scores, and rated as excellent, good, average and poor in each patient.^15^,^16^ One dental surgeon performed the oral health status evaluation throughout the study.

This study was approved by the Ethics Committee of the Nelson R Mandela School of Medicine, University of Natal.

## The frequency of bacteraemia following dental extraction

In this part of the study, only one tooth was extracted per patient. The same dental surgeon performed the procedure using dental forceps; no surgical procedures were used in any patient.

The skin at the site of the venepuncture was prepared using 0.5% chlorhexidine in 70% alcohol. Using standard aseptic techniques, 8–10 ml of blood was drawn immediately prior to and at two, five, 15 and 30 minutes after the extraction in each patient.

Three to 5 ml of blood was injected directly into BACTEC (Becton Dickinson, Maryland, USA) blood culture vials type 6b (aerobic) and 7d (anaerobic), respectively, after the used needle was replaced with a new, sterile needle and the rubber septum on the BACTEC vials was disinfected with alcohol.

The blood culture bottles were transported to the Microbiology Department, King Edward VIII Hospital, Durban within two hours of collection and were immediately incubated at 37°C. In the case of the aerobic bottles, this also included agitation on BACTEC shakers for the first 24 hours.

The blood culture vials were tested on days one, three, five and seven, and positive vials were sub-cultured and Gram-stained smears were prepared. The aerobic vials were sub-cultured onto chocolate, blood and MacConkey agar plates, which were incubated for 48 hours in air plus 10% CO_2_. The anaerobic vials were sub-cultured onto 10% blood agar plates with and without amikacin, which were incubated for 48 to 72 hours in anaerobic gas pak (Becton Dickinson, USA) jars with appropriate controls.

The organisms isolated were further identified using conventional laboratory methods and the identity of streptococcal isolates was confirmed using the API Strep 20 (API, France) system.^17^

## The frequency of bacteraemia following tooth brushing

In this part of the study, patients were instructed on the proper technique of tooth brushing by the dental surgeon. Thereafter they brushed their teeth for about five minutes using a new soft toothbrush and toothpaste.

The skin preparation and the techniques for blood collection and blood culture were similar to the first part of the study. The timing of the blood sampling was immediately prior to, immediately after, and at five and 15 minutes after tooth brushing.

## The frequency of bacteraemia following chewing

In this part of the study, patients were asked to chew an apple. The skin preparation and the techniques for blood collection and blood culture were similar to the first part of the study. The timing of the blood sampling was immediately prior to commencement of chewing, when half the apple had been eaten, when the whole apple had been eaten, and five minutes later.

## Statistical analysis

In each part of the study, the patients with plaque and gingival index scores rated as excellent, good, average and poor were placed in their respective groups and the number of patients with positive blood cultures in each group was compared using the Chi-square test. A *p*-value < 0.05 was chosen as the level of significance.

## Results

### The frequency of bacteraemia following dental extraction

A total of 108 black patients participated in the study. There were 60 males and 48 females. Their ages ranged from 16 to 66 years (mean 29.5).

No patient had a plaque or gingival index score that was rated as excellent. One patient who had a plaque and gingival index score rated as poor had a bacteraemia prior to dental extraction. *Bacteroides fragilis* was detected on anaerobic culture.

Post-extraction bacteraemia was detected in 32 (29.6%) patients and was transient in all patients. No bacteria were detected at 15 or 30 minutes. The organisms cultured after dental extraction are listed in Table 1. The number of patients who had positive blood cultures in the groups with good, fair and poor plaque and gingival index scores are shown in Tables 2 and 3.

**Table 1.** Organisms Cultured After Dental Extraction

*Aerobic cultures*	*No.*	*Anaerobic cultures*	*No.*
*Streptococcus mitis*	5	*Streptococcus mitis*	5
*Streptococcus sanguis*	4	*Streptococcus sanguis*	1
*Streptococcus anginosus* group	4	*Streptococcus anginosus* group	4
*Streptococcus* species	6	Viridans streptococci	1
*Staphylococcus epidermidis*	1	*Streptococcus* species	5
*Enterococcus faecalis*	2	*Staphylococcus epidermidis*	2
*Moraxella catarrhalis*	1	*Enterococcus faecalis*	1
*Neiserria sicca*	1	*Moraxella catarrhalis*	1
*Corynebacterium ulcerans*	1	*Neiserria sicca*	1
*Corynebacterium xerosis*	1	*Corynebacterium ulcerans*	1
		*Corynebacterium xerosis*	1
		*Prevotella melaninogenica*	1
		*Capnocytophaga* species	1
		Gram-negative bacilli	1

**Table 2.** Patients With Positive Cultures After Dental Extraction In Relation To Plaque Index*

*Plaque index score*	*No. in group*	*No. positive*	*% positive*
Good	36	10	27.8
Fair	37	12	32.4
Poor	35	10	28.6

*Differences between the groups were not statistically significant.

**Table 3.** Patients With Positive Cultures After Dental Extraction In Relation To Gingival Index*

*Gingival index score*	*No. in group*	*No. positive*	*% positive*
Good	38	9	23.7
Fair	34	12	35.3
Poor	36	11	30.6

*Differences between the groups were not statistically significant.

## The frequency of bacteraemia following tooth brushing

Seventy-four black patients, 39 males and 35 females, entered the study. Their ages ranged from 16 to 63 years (mean 26.6). No patient had a plaque or gingival index score that was rated as excellent.

No bacteraemia was detected prior to tooth brushing. Bacteraemia was detected in eight (10.8%) patients after tooth brushing. The duration was less than 15 minutes. The organisms cultured after tooth brushing are listed in Table 4. The number of patients who had positive blood cultures in the groups with good, fair and poor plaque and gingival index scores are shown in Tables 5 and 6.

**Table 4.** Organisms Cultured After Tooth Brushing

*Aerobic cultures*	*No.*	*Anaerobic cultures*	*No.*
*Streptococcus sanguis*	2	*Streptococcus sanguis*	2
*Streptococcus salivarius*	1	*Streptococcus salivarius*	1
*Viridans* streptococci	2	*Bacillus* species	1
		*Corynebacterium* species	1

**Table 5.** Patients With Positive Cultures After Tooth Brushing In Relation To Plaque Index*

*Plaque index score*	*No. in group*	*No. positive*	*% positive*
Good	24	2	8.3
Fair	24	1	4.2
Poor	26	5	19.2

*Differences between the groups were not statistically significant.

**Table 6.** Patients With Positive Cultures After Tooth Brushing In Relation To Gingival Index*

*Gingival index score*	*No. in group*	*No. positive*	*% positive*
Good	24	2	8.3
Fair	25	2	8
Poor	25	4	16

*Differences between the groups were not statistically significant.

## The frequency of bacteraemia following chewing

There were 32 black patients, 20 males and 12 females, in this part of the study. Their ages ranged from 16 to 45 years (mean 25). Both the plaque and gingival index scores were rated as good, fair and poor in 11, 12 and nine volunteers, respectively.

None of the blood cultures taken before, during and following mastication yielded any bacterial growth. Based on this preliminary analysis, it was decided to terminate the study. The target number in this part of the study was 60 patients.

## Discussion

There are conflicting data regarding the degree of oral disease necessary to produce bacteraemia after oral procedures. Okell and Elliott found that the occurrence and degree of bacteraemia after dental extraction depended upon the severity of gum disease,^7^ whereas McEntegart and Porterfield found that the incidence of post-extraction bacteraemia was unrelated to the extent of oral sepsis.^18^ In children, Peterson and Peacock found that the incidence of bacteraemia following dental extraction was unrelated to the local disease,^19^ while Speck *et al*. found that bacteraemia was more common after extraction of abscessed teeth.^20^

Cobe reported that the occurrence of periodontal disease had little effect on the incidence of bacteraemia after tooth brushing,^21^ whereas the data of Sconyers *et al*. suggested that there was a relationship between the frequency of bacteraemia after tooth brushing and oral health.^22^,^23^ Cobe also found no relationship between periodontal disease and the occurrence of bacteraemia after chewing.^21^

Attempts to establish a relationship between bacteraemia and oral disease from published reports have been extremely difficult. The first problem relates to terminology. Cooke noted that it was only in the 1960s that the all-embracing term ‘oral disease’ was replacing the older and equally vague term ‘oral sepsis’ and that these had clouded the interpretation of any relationship between specific oral disease such as gingivitis and periodontitis, and bacteraemia.^24^ Furthermore, terms such as ‘dental sepsis’ and ‘moderate’ and ‘severe gum disease’ were not defined.

The second problem relates to the diversities in the type of surgical procedures (e.g. single vs multiple dental extractions), time of blood sampling, volume of blood cultured and the methods used to isolate and identify the micro-organisms. This hindered interpretation and comparison of the results.^12^

In our study, we were unable to find a relationship between the plaque and gingival index scores, which are indicators of oral health status, and bacteraemia following dental extraction. Bacteraemia occurred after dental extraction in 27.8, 32.4 and 28.6% of black patients with good, fair and poor plaque index scores, and in 23.7, 35.3 and 30.6% of patients with good, fair and poor gingival index scores, respectively. These differences were not statistically significant.

Coulter *et al*. found that there was no relationship between the incidence or the intensity of post-extraction bacteraemia and either the amount of plaque around the gingival margin or the gingival condition in children.^25^

Lewis *et al*. reported that there was no correlation between oral health status and positive blood cultures after dental extraction in black patients in the abstract of their article.^26^ However, in their discussion they stated that although the majority of their subjects were considered to have poor oral health, it was not possible to draw any definite conclusion about the relationship between oral health and post-extraction bacteraemia. Lockhart^27^ and Lockhart *et al*.^28^ found that the degree or severity of oral disease did not correlate with the results of blood culture.

In our study, 29.6% of our patients developed bacteraemia after dental extraction. The frequency of positive blood cultures after dental extraction ranged from zero to 85% (mean 40%).^8^ Using a lysis-filtration technique to process blood samples, Heimdahl *et al*. observed bacteria in 100% of patients after dental extraction.^29^ Using molecular techniques, Lockhart *et al*. found that the cumulative incidence of infective endocarditis-related bacteraemia was 60.4%.^28^

Lewis *et al*. studied 60 black patients and detected bacteraemia in 65% of patients after dental extraction.^26^ Streptococci accounted for 35.7% of their positive blood cultures. In our study, streptococci made up 76.9% of the bacteria isolated. These data are in keeping with published reports, which indicate that viridans streptococci are the most frequent micro-organisms cultured after extraction.^5^,^12^

None of our patients had positive blood cultures at 15 and 30 minutes after extraction. The duration of bacteraemia after dental extraction is relatively brief, less than 30 minutes.^8^ Some investigators have found blood cultures positive for organisms further on in time but they drew blood specimens following surgical procedures of different durations.^27^

In our study, tooth brushing produced bacteraemia in 10.8% of black patients and the duration was less than 15 minutes in all patients. Bacteraemia was detected in the studies by Cobe (24.2% positive),^21^ Rise *et al*. (26.0% positive),^30^ Schlein *et al*. (25% positive),^31^ Roberts *et al*. (38.5% positive),^2^ Sconyers *et al*. (16.1% positive),^22^ Kinane *et al*. (3%)^32^ and Forner *et al*. (10%)^33^ but not in the studies by Sconyers *et al*.,^23^ Berger *et al*.^34^ and Hartzell *et al*.^35^ Streptococci were the predominant organisms isolated in these studies as well as in our study.

In our study, bacteraemia was unrelated to oral health status. The frequency of bacteraemia in the study by Bhanji *et al*. was 46%.^36^ They found no correlation between the plaque and gingival scores and the occurrence of bacteraemia. However, using molecular techniques, Lockhart *et al*. reported that the cumulative incidence of infective endocarditis-related bacteraemia was 22.5% and that the incidence was significantly related to the state of oral hygiene and gingival disease parameters.^28^

Chewing did not produce bacteraemia in our study. Similar results were obtained by Robinson *et al*.^37^ in patients chewing wax, and by Cobe^21^ in patients chewing gum. On the other hand, bacteraemia was detected after chewing bubble gum in 22% of patients by Diener *et al*.,^38^ after chewing paraffin in 55% of patients by Murray and Moosnick,^39^ after chewing hard candy in 17.4% of patients by Cobe,^21^ and after chewing bubble gum in 20% of patients by Forner *et al*.^33^

One patient in our study had a bacteraemia prior to dental extraction. His plaque and gingival index scores were rated as poor. *Bacillus fragilis* was isolated. Reith and Squier obtained positive blood cultures from 12% of 99 patients with no demonstrable focus of infection.^40^ Okell and Elliott found that 10.9% of 110 patients with pyorrhoeal disease had a streptococcal bacteraemia before any operative procedures.^7^ Rogosa *et al*.^41^ and Roberts *et al*.^2^ found positive blood cultures unrelated to dental procedures.

In a study of different antibiotic regimens in the prevention of bacteraemia after dental extraction , Diz Dios *et al*. reported that positive blood cultures were present prior to the dental extraction in all four of their study groups (range: 5–12.5%).^42^ On the other hand Cobe,^21^ Peterson and Peacock,^19^ and Hall *et al*.^43^ found no growth in pre-procedure blood cultures.

Our study confirmed that bacteraemia occurs after tooth brushing. In the past, emphasis was placed on antibiotic prophylaxis prior to dental procedures, especially dental extraction.

Only 4% of the 1 322 patients with infective endocarditis studied by Guntheroth had extractions in the previous two months.^8^ He noted that bacteraemia occurred in 40% of patients after dental extraction, in 25% after tooth brushing or oral irrigation, and in 38% with normal chewing. He concluded that in a hypothetical month ending with a dental extraction, the number of exposures to bacteraemia is almost 1 000 times more for ‘physiological sources’ (e.g. tooth brushing and chewing) than from a dental extraction. He stated that the physiological sources of bacteraemia would explain the occurrence of endocarditis due to viridans streptococci in patients who did not have dental extractions.

Roberts also reported that the cumulative exposure to bacteraemia from everyday procedures such as tooth brushing was significantly greater than that following invasive procedures such as dental extraction.^44^

## Conclusion

The emphasis in the prevention of infective endocarditis has now shifted from the use of antibiotics prior to dental procedures to the maintenance of good oral health in patients at risk of developing infective endocarditis.^45^,^46^
